# Surprises From the Basal Ganglia: Stop and Go Have New Meaning

**DOI:** 10.1002/mds.70008

**Published:** 2025-08-14

**Authors:** Ann M. Graybiel

**Affiliations:** ^1^ McGovern Institute for Brain Research and Department of Brain and Cognitive Sciences Massachusetts Institute of Technology Cambridge Massachusetts USA

**Keywords:** striatum, canonical direct and indirect basal ganglia pathways, striosome, pallidum, Parkinson's disease, mood and movement control

## Abstract

This perspective highlights new work suggesting the need for revision of the canonical direct–indirect model of the basal ganglia's influence on movement, with fresh evidence that there is a formerly unappreciated pair of direct and indirect pathways that parallel the standard model's canonical direct and indirect pathways, and promising evidence pointing toward improved clinical treatments for Parkinson's disease. As a working hypothesis, it is suggested that the non‐canonical direct and indirect pathways, which arise in striosomes, might act as homeostatic circuits that can reign in or amplify the activity of the canonical pathways in the face of their imbalance, including that occurring in hyperkinetic or hypokinetic disorders. © 2025 The Author(s). *Movement Disorders* published by Wiley Periodicals LLC on behalf of International Parkinson and Movement Disorder Society.

For nearly 40 years, the most important clinical model of movement disorders involving the basal ganglia has been that there are two great opposing neural circuits, known as the direct and indirect pathways (nicknamed the Go and No‐Go pathways) whose balance in activity is necessary for normal motor control, and whose imbalanced activity can lead to hypokinetic or hyperkinetic disorders. Premier examples include Parkinson's disease (PD) and Huntington's disease (HD). A persistent uncertainty, however, has been how to account for non‐motor symptoms that, as discussed frequently in this Journal's pages, can occur, including those affecting cognition and mood states. Controversies around the direct–indirect model have also steadily accumulated. Here I lay out surprising new findings from experimental work that bear directly on and help to clarify these issues. Because the work is fresh and still in progress, I begin with the caveat that there is much more to learn.

## 1 The Canonical Direct–Indirect Control System of the Matrix Compartment

It is useful to start with the classic view of this circuit organization, which is that there are two great output pathways of the basal ganglia: a direct pathway from striatal projection neurons expressing D1 dopamine (dSPNS) that acts to promote movement, and an indirect pathway whose SPNs (iSPNs) express D2 receptors that has an anti‐movement function.^1^, ^2^, ^3^, ^4^ This direct‐D1/indirect‐D2 model has been bolstered by much clinical work, inspired deep brain stimulation (DBS) protocols and pharmacological treatment design, and is supported by many experimental findings as well.

This bedrock model has survived many challenges, only to emerge with new interpretations, important corrections, and a range of previously unrecognized potential means of behavioral control (reviewed in Cox and Witten^5^ and Graybiel and Matsushima^6^). Early on, the ‘rate vs. pattern’ controversy called into question neuronal spiking rates as being the sole relevant determinants of activity in the pathways.^7^ Then the addition of concomitant oscillatory local field activity to the story, with evidence for gamma‐band pro‐movement and beta‐band anti‐movement, added a powerful perspective that has influenced DBS protocol design and guided electrocorticogram recordings.^8^ Then experimental evidence in mice demonstrated that SPNs giving rise to the direct and indirect pathways (dSPNs and iSPNs) can be simultaneously active, as though cooperating, not opposing one another as originally thought (eg, Cui et al.^9^ and Jin et al.^10^), and that they can function selectively (eg, Nonomura et al.^11^), and that they may represent reinforcement‐based policy rather than actions designated simply as Go or No‐Go.^12^ Years before the development of the experimental techniques that allowed this discovery, Mink and Thatch^13^ and Mink^4^ had already proposed that the direct pathway could serve to facilitate an intended movement, while the indirect pathway simultaneously inhibited competing movements. This prescient idea has resurfaced in a variety of contemporary forms. Evidence now even suggests that in‐transit direct pathway fibers (at least in the mouse) give off collaterals to most of the neurons of the external pallidum (GPe) that project back to the striatum,^14^, ^15^ potentially allowing their further Go/No‐Go coordination or, particularly interesting, making this conditional.

## 2 The Canonical Direct–Indirect Pathways and Reinforcement Learning

During these years, both electrophysiological recordings from the dopamine‐containing neurons of the substantia nigra and theoretical work on reinforcement learning (RL) led to the startling realization that the firing rates of many of the dopamine neurons fitted the roles of RL learners, developing reward prediction errors during the course of behavioral learning (reviewed in Cox and Witten^5^). Integration of the direct–indirect models with models of reinforcement learning was catalyzed by a classic series of studies by Kreitzer and colleagues.^15^, ^16^ They found in mice that optogenetic stimulation of dSPNs was positively reinforcing, whereas stimulation of the iSPNs was aversive. This was for many a welcome step, as it began to integrate the strong concurrence of RL models and dSPN–iSPN activities. But it also presaged the addition of behavioral influences indicating potential motivational drive to the exclusively motor control view of the circuits. The conditional nature of the functions of these pathways was further emphasized by the Witten group's demonstration that these presumed pathway‐specific functions can be changed, even reversed, by learning and decision‐making.^17^ These findings find clear resonance with studies of the striatum, indicating wholesale changes in patterning of striatal neurons as a result of learning and habit formation requiring decision‐making.^18^, ^19^, ^20^, ^21^ Work on the basal ganglia, including clinical work, needs to engage this neuroplasticity maximally as a therapeutic tool.

The concomitant discovery of the ‘hyper‐direct pathway’ (neocortex to the subthalamic nucleus, the final intermediary in the indirect pathway) was a crucial reinforcing discovery,^22^, ^23^ as it turned out to be a fast‐track ally of access to the thalamus and then frontal neocortex, bypassing the striatum altogether. More and more it looks like the striatum is specialized for learning, notably, but not exclusively, reinforcement‐based learning. Few data are available from interventional work in humans, but the fact this plasticity of the SPNs' activity profiles affects their outputs via the direct and indirect pathways is a crucial realization when therapies are to be designed.

## 3 The External Pallidal Segment as a Controller of Movement

An unexpected turn of events has been the increased focus on the formerly almost neglected nucleus that is the intermediary stop in the classical indirect pathway, the external segment of the globus pallidus, the GPe. This nucleus turns out to be a hotbed of local and input–output circuity instrumental in behavioral control and to be a critical actor that can operate independent of its input from the indirect pathway's initiation site in striatal iSPNs.

**FIG. 1:**
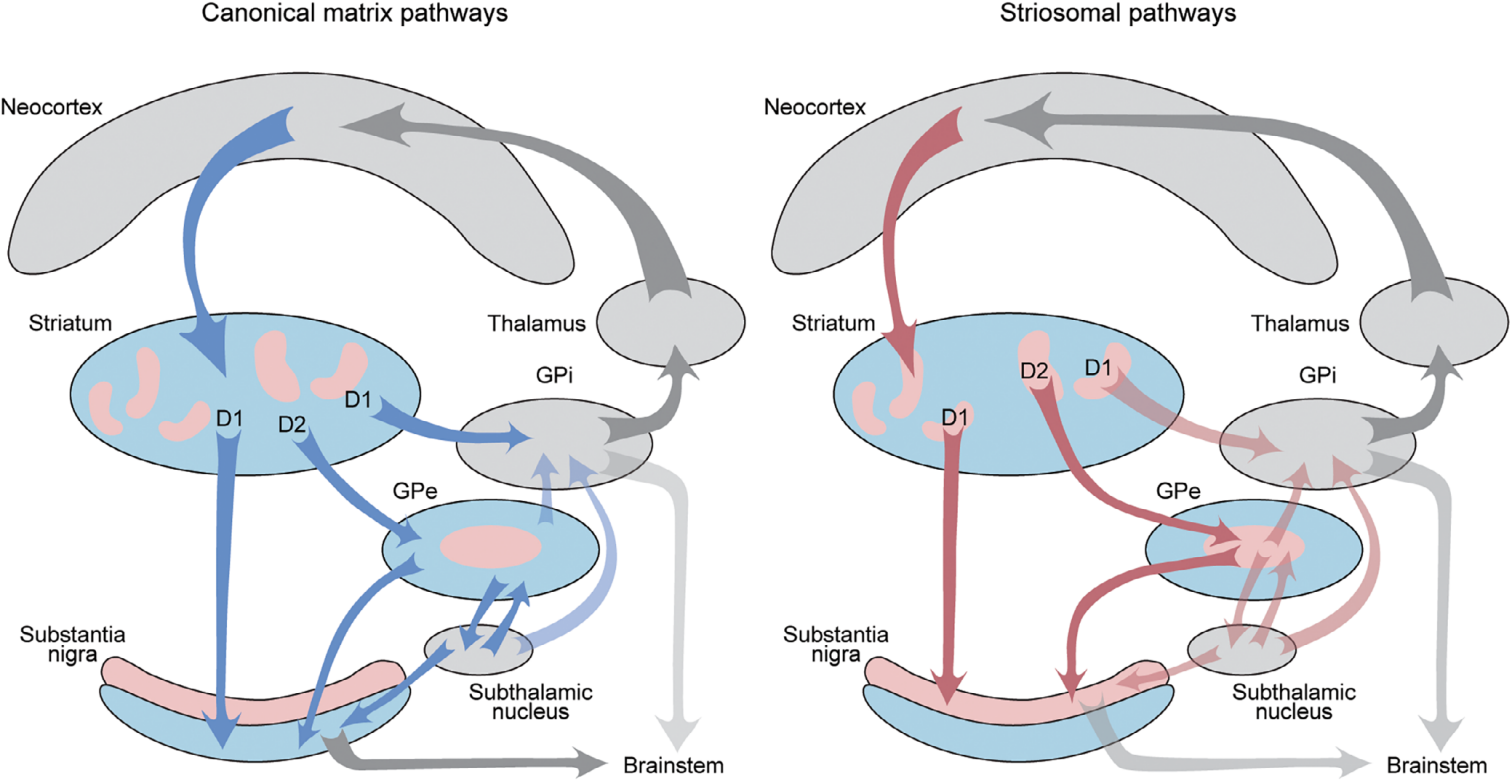
Schematic diagrams of the canonical matrix (left) and striosomal (right) direct and indirect pathways of the basal ganglia. In both diagrams, the striatal matrix compartment is shown in blue, and the striatal striosome compartment is indicated in pink. The arrows follow the same color scheme, as do the colors of their output targets. On the left, the canonical direct‐D1 pathway is shown to include the classic circuit from striatal D1‐expressing spiny projection neurons (dSPNs) of the matrix to the internal globus pallidus (GPi) (and substantia nigra pars reticulata [SNpr]) with continuation to thalamocortical and brainstem output targets. The canonical indirect‐D2 pathway includes iSPNs of the matrix and their projections to the external pallidum (GPe), GPe to subthalamic nucleus, and subthalamic nucleus to GPi (and SNpr) and the GPi to thalamocortical and brainstem targets. Also shown are subthalamic‐GPe loops. Note that the canonical D2 pathway largely avoids the pink part of the GPe, called the central zone in mice. We await identification of this zone in primates. The right‐hand diagram illustrates elements of the newly delineated striosomal direct‐D1 (S‐D1) and indirect‐D2 (S‐D2) pathways delineated in mouse experiments^24^; as yet incompletely verified links are indicated by pale arrows. The pink central zone within the GPe is the target of the S‐D2 circuit. Note that the S‐D1 and S‐D2 pathways mimic the canonical direct and indirect pathways but largely originate in the striosome, not in the matrix, compartment of the striatum, and target the substantia nigra pars compacta (SNpc) within which lie the dopamine‐containing neurons of the SN. Note that the cortical inputs to parts of the striosomal system, schematically shown by the reddish arrow, target striosomes; this is true for some limbic‐related cortical regions (eg, the pregenual anterior cingulate cortex and the caudal orbitofrontal cortex). By contrast, cortical inputs to the canonical pathways (schematically shown with blue arrows) largely include sensorimotor and association areas, and target zones within the matrix. Many other circuits are omitted for clarity (eg, prominent thalamic projections).

Originally, the terms direct and indirect were based on anatomy: the direct pathway dSPNs project directly to the internal globus pallidus (GPi), initiating the pallido‐thalamo‐cortical system (and the pallido‐nigro‐thalamo‐cortical system). But the iSPNs do not do this. They project to the GPe, which projects to the subthalamic nucleus, which projects to the GPi. The dSPNs and iSPNs are GABAergic and inhibit their targets (potential actions of their peptide co‐modulators are not considered here). There are complex internal connections of the globus pallidus, and a major GPi projection to the lateral habenula, also not considered in this brief review but well summarized elsewhere.^4^ Only the subthalamic nucleus has a preponderance of excitatory neurons. So the way this all would work out, according to the classical view of the direct–indirect model,^1^ here simplified for clarity, is that there is a double inhibition in the direct pathway: the striatum inhibits the pallidum, which inhibits the thalamus, so the net effect would be excitation of the thalamus. And for the indirect pathway, there is also inhibition (striatum to pallidum), but the pallidum then inhibits the subthalamic nucleus, disinhibiting the subthalamic projection to the pallidum, thus exciting inhibition of the thalamus, so that the classical view was that the net effect would be inhibition of the thalamocortical circuitry—hence the Go/No‐Go model (see Fig. 1).

Gittis and her colleagues, in a pivotal line of work in mice,^5^, ^25^, ^26^, ^27^ have challenged the classically interpreted motor effects of indirect pathway stimulation, that is, that iSPNs drive inhibition of movement (No‐Go) by virtue of their inhibition of the GPe. The Gittis group shows that direct inhibition of GPe (mimicking input from the iSPNs) does not reduce movement (locomotion of the mice), but rather produces a transient punishment effect, so that the mice will avoid a place where the inhibition is applied. They argue that the motor inhibition effect of stimulating the indirect pathway iSPNs comes from their inhibition of dSPNs by powerful local iSPN collaterals inhibiting the dSPNs.

Potential therapeutic treatment for PD patients is likely to come from this ongoing work: Gittis and her group have shown that cell‐type‐specific manipulations of genetically identified neurons in the GPe (excitation of parvalbumin neurons and inhibition of LHX6 neurons) can for sustained periods of time relieve the motor deficits in dopamine‐depleted parkinsonian model mice independent of the striatum. When non‐cell‐type‐specific stimulation is used, these beneficial effects do not occur. Specific stimulation parameters are also important—bursts of stimulation are needed. These findings have now led to the development of trials in humans.

The GPe thus turns out to be the epitome of a waystation that is not just passively sending inputs on to output targets but is itself deterministic in controlling function.

## 4 Non‐Canonical Direct and Indirect Pathways: Striosomes Targeting Dopamine‐Containing Neurons of the Substantia Nigra, Pars Compacta

The work just reviewed, collectively considered, could suggest that we should abandon the direct–indirect model altogether. But new work^24^ demonstrates that the fundamental direct–indirect architecture of the basal ganglia is echoed by a parallel set of direct–indirect pathways that had not before been recognized as such, but that likely are crucially important for the control of motivated behavior, possibly contributing to the non‐motor symptoms of PD and HD disorders. The finding of this repeat of the direct–indirect architecture tells us that this is likely to have been an evolutionarily important design, and that novel models of the basal ganglia are now required.

The canonical direct and indirect pathways originate in the large matrix compartment of the striatum, which houses most of the iSPNs and dSPNs and interneurons of the striatum. The newly recognized parallel set of direct and indirect pathways arises, instead, from the striosome compartment of the striatum, a labyrinthine system winding through the matrix and making up some 20% of the striatal volume. What is remarkable is that the striosomal direct and indirect pathways do not target the motor output nuclei of the striatum, as do the canonical direct and indirect pathways arising from the matrix. Instead, they target dopamine‐containing neurons of the substantia nigra pars compacta (SNpc), including the ventral tier of nigral neurons that is especially vulnerable in PD.^28^, ^29^ This means that the striosomes have access to the very neurons that must be therapeutically rescued in PD and that, under conditions of health, are thought to modulate both mood and movement. Whereas the matrix compartment receives large fractions of its inputs from sensorimotor and associative cortical regions, parts of the striosomal system are known to receive differentially strong inputs from regions of the neocortex linked to the limbic system, including the pregenual anterior cingulate and caudal orbitofrontal cortical regions as identified in non‐human primates (NHPs).^30^, ^31^, ^32^ These regions have been implicated in motivationally challenging approach‐avoidance decision‐making by experiments in both NHPs and, for their putative analogues, rodents.^31^, ^33^, ^34^, ^35^ These striosome‐projecting regions are hotspots for producing increased avoidance responses in situations requiring either accepting (approaching) or rejecting (avoiding) a given outcome, thus requiring subjective evaluations to guide action.

Striosomes have other functionally relevant features quite distinct from those of the matrix. Critical among these are different gene expression patterns^36^ and levels of many of the neurochemicals found in the striatum, for example, differentially enriched striosomal expression of mu opioid, serotoninergic 5HT2 receptors, cannabinoid CB1 receptors, and several neuropeptides; and there are contrasts as well in dopamine‐ and acetylcholine‐related compounds, and others.^37^ These distributions are also governed by gradients, so that not all regions of the striatum are the same for either compartment; nor are their functions. But these findings put the striosomal system in a specialized position with respect to potential actions of many psychoactive drugs and endogenous ligands. They also raise the possibility that these circuits are involved in the non‐motor symptoms of basal ganglia disorders such as PD and HD. The striosomal and matrix SPNs, in addition, have different birthdates and developmental progression rates. They thus potentially have different functions—and vulnerabilities—across prenatal and early postnatal development.^6^

It is remarkable that, despite their many differences between the striosome and matrix compartments, the fundamental design motifs of the matrix‐derived and striosome‐derived sets of direct and indirect pathways are similar, namely, that the D1 pathways of both have direct access to their targets and the D2 pathways of both have interrupted, therefore indirect, access to their targets. This commonality occurs despite the striking difference in their targets.

Just as the canonical direct pathway derives from D1‐expressing matrix dSPNs with direct monosynaptic access to their motor targets (GPi and substantia nigra pars reticulata, SNpr), the striosome‐derived direct pathway is made up of D1‐expressing striosomal dSPNs with direct monosynaptic access to their targets, the nigral dopamine‐containing neurons. This striosomal D1 (S‐D1) input is notable in contributing to the input side of “striosome‐dendron bouquets”, targeting and entwining themselves with bundled descending dendrites of the bouquets' dopamine‐containing neurons.^38^ The striosomal inputs, as shown in vitro in acute slice preparations,^7^, ^39^ can fully block the electrical activity of the bouquets' dopamine neurons and produce a subsequent large rebound excitation. Because the striosomal fibers target the descending dopamine‐containing dendrites, one possibility now suggested by ongoing in vivo experiments is that the striosomal S‐D1 pathway inputs affect dopamine release in the SNpc. The GPe projections to the SNpc tend to terminate more dorsally within the SNpc.^40^

For the indirect pathways, just as the canonical matrix‐derived indirect pathway derives from D2‐expressing iSPNs targeting the GPe, the striosomal indirect pathway derives from D2‐expressing iSPNs that target the GPe. This striosomal D2 (S‐D2) pathway, however, targets a special zone of the GPe that in mice is in a central part of the GPe (“central zone”) largely separate from the surrounding GPe regions targeted by the canonical indirect pathway input.^24^ Very little is known about this central zone, and its equivalent in primates is not yet known.

The first study of the S‐D1 and S‐D2 pathways in freely behaving mice has produced a striking result.^24^ The striosomal direct and indirect pathways have opposite effects to those of the canonical (matrix) direct and indirect pathways on both movement and dopamine release in the striatum. Optogenetic stimulation of the direct S‐D1 striosomal pathway (in particular, experimentally tagged D1 dSPNs) reduces movement instead of exciting movement like the canonical direct D1 pathway does, and decreases dopamine release in the striatum. Stimulation of the S‐D2 pathway (experimentally tagged) increases movement rather than reducing it. These results were obtained in mice freely moving in an open field and also as mice performed a decision‐making task in a T‐maze.^24^ Yet studies of learning and reinforcement effects are still to be done, leaving open the important question of how these limbic‐related S‐D1 and S‐D2 striosomal pathways affect motivation to act and, as well, questions about their potential differences in regions of the striatum.

What could be the value of such a parallel organization? One possibility is that the striosomal system provides a homeostatic modulation of the canonical pathways under the influence of cortical and other inputs related to the demands of cognition and subjective evaluation. If the canonical direct pathway develops excessive activity, producing excessive, even meaningless movement as can occur in some basal ganglia disorders, the striosomal S‐D1 direct pathway could apply a dampening down of the activity via inhibition of the dopamine neurons. Notably, striosomes have been found to be differentially vulnerable in the HD striatum as examined postmortem in a small sample of brains from patients identified as having had disorders of mood.^41^, ^42^ For the indirect pathway system, an analogous homeostatic function could be to energize action in the face of excessive drive from the canonical D2 indirect pathway via disinhibition of the dopamine‐containing neurons.

What these findings introduce here is the concept that striosome‐based direct–indirect circuits, engaging mood‐related neocortical and subcortical regions, could affect behavioral state under conditions that include those with risk, uncertainty, and motivational challenge—mood changes exhibited by many PD and HD patients. Additional studies at many levels clearly are needed—and even with the many experimental new tools now available, they will be difficult. But it is not too early to point out that the S‐D2 pathway likely has been unknowingly affected by clinical treatment with D2 drugs given for many purposes, including for the treatment of PD. Learning more about this remarkable duality of direct and indirect basal ganglia systems should thus be valuable clinically as well as for basic understanding of motor and mood control by the forebrain.

## Author Roles

(1) Manuscript Preparation: A. Writing, B. Reference Compilation, C. Figure Design.A.M.G.: 1A, 1B, 1C.

## Financial Disclosures of All Authors (for the Past 12 Months)

This work was funded by the National Institutes of Health (NIH)/National Institute of Mental Health (NIMH) (R01 MH060379), CHDI Foundation (A‐5552), William N. and Bernice E. Bumpus Foundation, Saks‐Kavanaugh Foundation, and Jim and Joan Schattinger.

## Data Availability

Data sharing is not applicable to this article as no new data were created or analyzed in this study.
